# Estrogenic Potency of Benzophenone UV Filters in Breast Cancer Cells: Proliferative and Transcriptional Activity Substantiated by Docking Analysis

**DOI:** 10.1371/journal.pone.0060567

**Published:** 2013-04-04

**Authors:** Gwenneg Kerdivel, Remy Le Guevel, Denis Habauzit, François Brion, Selim Ait-Aissa, Farzad Pakdel

**Affiliations:** 1 Transcription, Environment and Cancer Group, Institut de Recherche sur la Santé, Environnement et Travail (IRSET), INSERM U1085, Université de Rennes 1, Rennes, France; 2 ImPACcell, SFR Biosit, Université de Rennes 1, Rennes, France; 3 In Vitro and In Vitro Ecotoxicology Group, Institut National de l'Environnement Industriel et des Risques (INERIS), Parc Technologique Alata, Verneuil-en-Halatte, France; Massachusetts General Hospital, United States of America

## Abstract

The results from recent studies show that some benzophenones (BPs) and their hydroxylated metabolites can function as weak estrogens (E2) in the environment. However, little is known about the structure-activity relationship of these molecules. We have examined the effects of exposure to ten different BPs on the proliferation of estrogen receptor (ER)-positive breast cancer cells and on the transcriptional activity of E2-target genes. We analyzed two genes that are tightly linked with estrogen-mediated proliferation, the CXCL12 and amphiregulin genes and two classical estrogen-responsive genes, the pS2 and progesterone receptor. Significant differences in the BPs efficiency to induce cell proliferation and endogenous E2-target gene expressions were observed. Using ERE-, Sp1-, AP1- and C3-reporter genes that contain different ER-binding sites in their promoter, we also showed significant differences in the BPs efficiency in activation of the ER transactivation. Together, our analyzes showed that the most active molecule is 4-hydroxy-BP. Docking analysis of the interaction of BPs in the ligand-binding pocket of ERα suggests that the minimum structural requirement for the estrogenic activity of BPs is a hydroxyl (OH) group in the phenyl A-ring that allows interaction with Glu-353, Arg-394 or Phe-404, which enhances the stability between BPs and ERα. Our modeling also indicates a loss of interaction between the OH groups of the phenyl B-ring and His-524. In addition, the presence of some OH groups in the phenyl B-ring can create repulsion forces, which may constrain helix 12 in an unfavorable position, explaining the differential estrogenic effects of BPs. These results, together with our analysis of BPs for their potency in activation of cell proliferation and ER-mediated transcription, report an improved understanding of the mechanism and structure–activity relationship of BPs.

## Introduction

During the past years, scientific works have reported a growing concern with the increase in emerging environmental contaminants and their potential impact on the ecosystem and human health [1].

**Figure 1:**
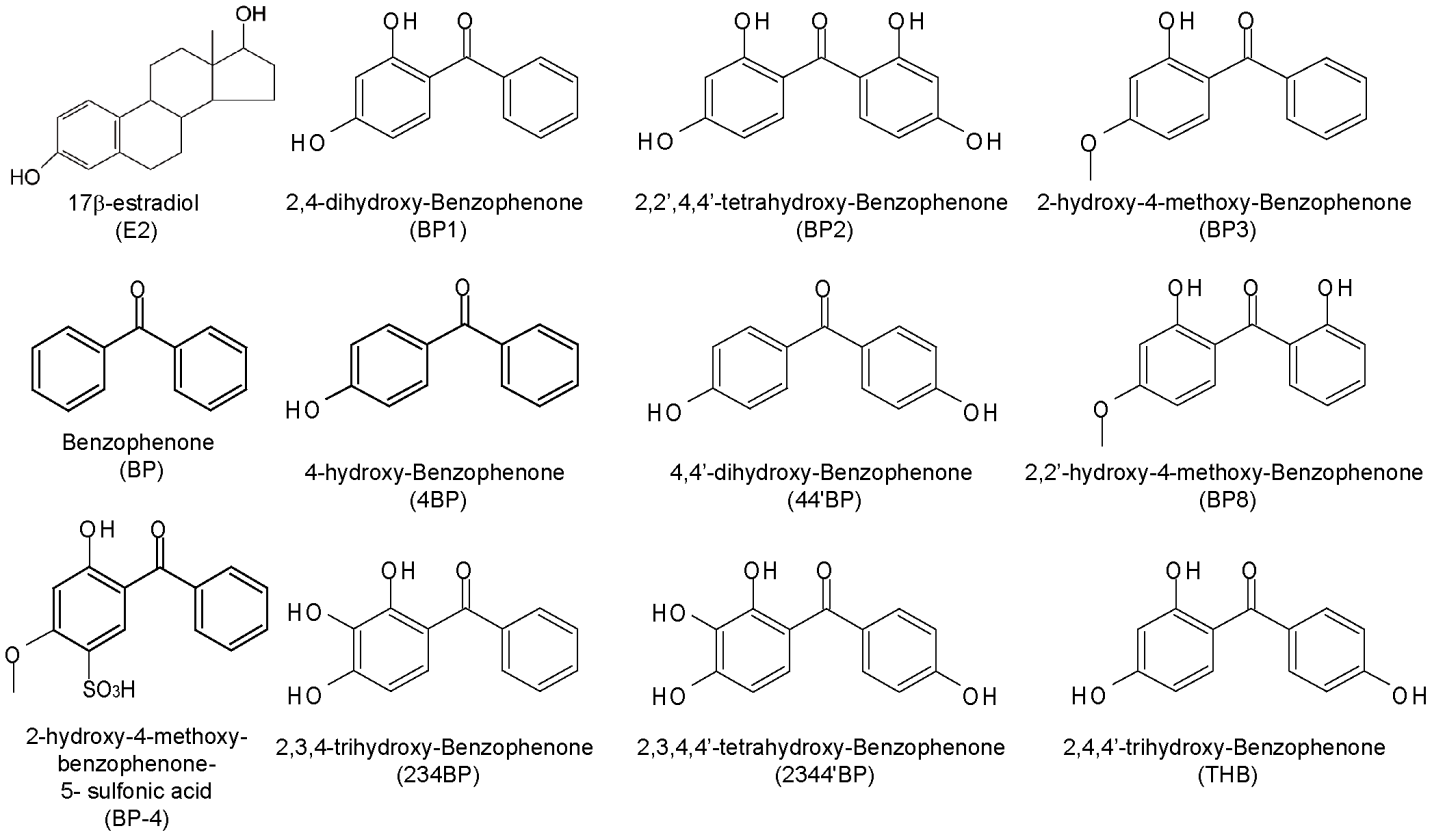
Chemical structures of 17β-estradiol, 2-hydroxy-4-methoxy-benzophenone-5sulfonic acid and the ten benzophenone derivatives analyzed in this study.

Benzophenones (BPs) are organic compounds with ultraviolet (UV) filter properties that absorb UV-A (315–400 nm) and UV-B (280–315 nm) radiation. These chemicals are largely used in cosmetic products, particularly sunscreens and skin lotions, but also as additives in plastics, printing inks, shampoos, perfumes and photographic films to prevent UV light damage. BPs have a relatively low molecular weight and contain two phenol rings with various hydroxyl (OH) groups (Fig. 1). The highly lipophilic properties of BPs enable them to rapidly cross the dermal tissue, which can cause bioaccumulation in the human body. A few hours after application, these BP UV filters could be detected in the plasma, bile and urine. Furthermore, some of the UV filters have been detected in human milk [2]. UV filters were also found in the surface water of lakes and rivers (Table 1), where they can be in direct contact with fishes and individuals or indirectly through wastewater treatment plants [3]. The concentration of some BPs in sewage sludge can exceed that of polychlorinated biphenyls, reaching 10 mg/kg of dry matter. For instance, BP-3 has been detected at levels between 60–125 ng/L in Swiss lake water and 123–1,800 ng/g in fish lipid from perch and roach [4]. BP-4, one of the most common UV filters in the aquatic environment in Switzerland [3], was also found at levels up to 849 ng/L in rivers and 1,481 ng/L in wastewater in Spain [5]. Thus, in addition to dermal absorption, humans might also be exposed to UV filters by eating contaminated fish or seafood.

**Table 1:** Concentrations of several BPs investigated in the environment and in human samples.

Matrix		BP	BP1	BP3	4BP	BP8	References
Water samples (ng/L)	Rivers	23	LOD	14	6	–	[43]
			LOD-24	LOD-87	–	–	[44]
	Lakes	–	–	5–125 (july)	–	–	[38]
	Sea water	–	LOD-280	1340–3300	–	LOD	[45]
	Raw wastewater	–	31–148	184–429	–	–	[44]
	Treated wastewater	–	LOD-13	LOD-84	–	–	[44]
Soil Samples (ng/g)	Sewage sludge	–	LOD	LOD-790	LOD-150	–	[46]
		–	4.41–91.6	2.05–13.3	2.66–10.1	LOD	[47]
	Sediment	–	0.259–0.607	0.728–4.66	0.312–0.951	0.133–0.796	[47]
		1.52–9.73	LOD	LOD	18.38	0.5–2.14	[48]
	Ground Soil	0.82–16.55	LOD	0.73–3.88	1.06–4.91	0.5–4.17	[48]
Human biological samples	Urine (ng/mL) (Women)	–	LOD-3200	LOD-5900	LOD-22	–	[42]
		–	–	1.3–22.9	–	–	[49]–[51]
	Urine (ng/mL)*	–	–	Women: 200/Men: 300	–	–	[52]
		–	–	Women: 187/Men: 238	–	–	[53]
	Plasma (ng/mL)*	–	–	Women: 60/Men: 140	–	–	[52]
		–	–	Women: 44/Men: 81	–	–	[53]
	Milk (ng/g lipid)	–	–	52.23±50.69	–	–	[54]

LOD: below the detection limit; *Maximum median concentration observed during 96 h exposure to 10% BP3-containing sunscreen with daily whole-body application.

BPs could be considered emerging environmental contaminants because their amounts are increasing in the environment and their activities are not yet well defined. The results of recent studies revealed that some of these molecules act as endocrine disrupting chemicals (EDCs). In fact, *in vitro* and *in vivo* studies in different species of mammals and fish showed that some of these UV filters exhibit hormonal activity and are able to interact with estrogen, androgen and thyroid signaling [6]–[8]. *In vivo* studies using the uterotrophic assay on immature rats as well as the vitellogenin assay in fish have shown that some of the UV filters act as weak estrogen mimics [2], [9]. Long-term treatment in rats showed that some of the UV filters also mimic the typical effects of 17-beta estradiol (E2), including fat deposits, lipid metabolism, and delay of puberty and decrease of prostate weight in exposed males [10]. Moreover, *in vitro* studies using fish and mammalian cell lines as well as fish and human estrogen receptors (ERs) expressed in yeast have reported direct estrogenic effects of some commonly used BP derivatives [9], [11]–[12]. These results have shown that the estrogenic potencies of these compounds are much lower (up to 10,000 fold) compared to E2 or the potent pharmaceutical estrogen ethynyl-estradiol. Moreover, competitive ER binding assays have shown that BPs compete with E2 binding at the ER ligand binding site. Although this finding confirmed the relatively low affinity of BPs for ERs, which was estimated to be 100-1,000 times lower than that of the natural ligand, these analyses suggested the direct actions of BPs *via* ERs.

ERs mediate the multiple cellular effects of estrogens by diverse transcriptional mechanisms that are the first steps toward cell phenotypic changes. In the classical pathway, ERs regulate the expression of E2-target genes by direct interaction with a specific DNA sequence in the promoter of E2-target genes, the Estrogen Response Element (ERE). This ER-DNA interaction permits the recruitment of the cofactors that are necessary for transcription. However, for a number of important E2-sensitive genes, which do not contain the ERE, ERs can also regulate transcription through protein–protein interactions with transcription factors already bound to the promoter, such as stimulating protein 1 (Sp1) or activator protein 1 (AP1) [13]–[15]. Furthermore, it is believed that ERs change from an inactive state to a transcriptionally active form through an allosteric ligand-inducing conformational change [16], [17]. The carboxy-terminal or ligand-binding domain of ERs is composed of 12 individual alpha helices (H1 to H12). In the ER-ligand complex, the ligand interacts with the ligand-binding pocket formed by helix H3, H4, H5 and H12. The precise positioning of H12 is essential for cofactor recruitment and transcriptional activity of the ERs. Thus, mechanisms governing the expression of E2-target genes and recruitment of specific cofactors involve both the promoter context and ER ligands.

Previous studies have reported estrogenic/anti-estrogenic activity as well as ERα/ERβ selectivity of some BP derivatives [11], [12], but their direct effects on structurally different E2-target genes have not been fully explored. Moreover, little is known about their potential impact in breast cancer cell growth. Thus, the assessment of estrogenic potencies of BPs in breast cancer cells in relation to the ER transactivation requires further analysis. In this study, we examined the effects of 10 BPs (Fig. 1) on the proliferation of breast cancer cells and on structurally different ER-target gene transcription in MCF-7 breast cancer cells. We found that BPs exhibit a differential activation of E2-target genes, including endogenous genes (CXCL12, amphiregulin, pS2 and Progesterone Receptor) and ERE-, SP1-, AP1- and C3-reporter genes. Our docking study by computer simulation of the interaction of BPs with the ligand binding pocket of ERα suggests that unlike E2, BPs do not interact with His-524, but only with Arg-394 and Glu-353 (H-bond interaction) and Phe-404 (π-π interaction). However, an alternative H-bond interaction with Thr-347 on helix 3 is observed with some B-ring hydroxylated BPs. Moreover, based on differences in the residues that interact with the ERα ligand-binding site, BPs could lead to slight ligand-dependent conformational changes of the activated receptor, which could alter its cofactor recruitments, transcriptional regulation and cell response. Altogether, our results highlight the necessity to perform multiple tests to precisely define the estrogenic potency of an environmental compound. Finally, in regards to the proliferative effects of BPs, it seems plausible to postulate a potential pro-carcinogenic effect of these molecules in ERα-positive tissues.

## Materials and Methods

### Antibodies and Reagents

17-β-estradiol (E2), ICI_182,780_ (ICI), benzophenone (BP), 2,4-dihydroxy-benzophenone (BP1), 2,2′,4,4′-tetrahydroxy-benzophenone (BP2), 2-hydroxy-4-methoxy-benzophenone (BP3), 4-hydroxy-benzophenone (4BP), 4,4′-dihydroxy-benzophenone (44′BP), 2,2′-hydroxy-4-methoxy-benzophenone (BP8), 2,3,4-trihydroxy-benzophenone (234BP), 2,3,4,4′-tetrahydroxy-benzophenone (2344′BP) and 2,4,4′-trihydroxy-benzophenone (THB) were acquired from Sigma-Aldrich Co (Fig. 1).

The primary antibodies used for Western Blot analysis were rabbit polyclonal antibodies against ERα (HC-20, sc-543) and mouse monoclonal antibody against β-actin (AC-15, sc-69879), acquired from Santa Cruz. The peroxidase-conjugated secondary antibodies used were a goat anti-rabbit purchased from Pierce and a goat anti-mouse from Santa Cruz.

### Cell culture and treatments

The MCF-7 human breast cancer cell lines were purchased from the American Type Culture Collection (Manassas, VA, USA). MCF-7 cells were maintained in Dulbecco Modified Eagle's Minimal Essential Medium (DMEM, Invitrogen) containing 10% fetal bovine serum (FBS, Sigma, St. Louis, MO, USA) and antibiotics (Invitrogen) at 37°C in 5% CO_2_. Steroid treatments were preceded with a 48 h hormone and serum-deprivation stage in DMEM-F12 (Sigma) supplemented with 2.5% dextran treated charcoal stripped FBS (dsFBS).

### Plasmids, transient transfections and Luciferase assays

Four luciferase reporter plasmids with different estrogen-sensitive promoters were employed in the transfection experiments: an artificial promoter containing one ERE upstream of the TK promoter (ERE-Luc) [18], the AP1-Luciferase (AP1-Luc) and Sp1-Luciferase (Sp1-Luc), which were obtained from Panomics (Panomics Inc, Fremont, CA), and the complement 3-Luciferase (C3-Luc) that was described previously [19]. A CMV-βgal expression vector (Promega) was used as an internal control.

The cells were transfected using JetPEI™ as described in the manufacturer's protocol (Polyplus Transfection™). Transfections were performed overnight with MCF-7 cells after 24 h of hormone depletion with 200 ng of reporter gene and 25 ng of CMV-βGal as an internal control. The cells were then treated with vehicle, E2 and BPs for 48 h. Next, the cells were lysed in Passive Lysis Buffer (Promega), and the luciferase activity was measured using a commercial kit (Promega). Each luciferase assay was performed in triplicate, and the result was reproduced in at least three independent experiments.

### RT-PCR assays

Total RNA extractions were performed using Trizol™ reagent (Invitrogen) according to the manufacturer's recommendations. cDNAs were obtained using MMLV reverse transcriptase (Promega). Quantitative RT-PCRs were performed on a BioRad MyiQ apparatus using the iQ™ SYBR® Green supermix from BioRad (BioRad, Hercules, CA, USA). The sequences of the primers used for amplification of cDNA in the RT-PCR experiments are: CXCL12, Rev: GCCTCCATGGCATACATAGG, Fwd: CTCCTGGGGATGTGTAATGG; Amphiregulin, Rev: CCTGGCTATATTGTCGATTCA, Fwd: GTATTTTCACTTTCCGTCTTGTTTTG; pS2, Rev: CCGAGCTCTGGGACTAATCA, Fwd: ACCATGGAGAACAAGGTGA; and PR, Rev: CCCGCCGTCGTAACTTTCG, Fwd: GTGCCTATCCTGCCTCTCAATC and GAPDH: rev: GAGGTCCACCACCCTGTTGC, fwd: GGGCATCCTGGGCTACACTG (Proligo Primers and Probes, Boulder, CO, USA).

### Proliferation assays

MCF-7 cells were plated in 96-well plates. After 48 h of hormone-deprivation, the cells were treated with vehicle, E2 (10^−14^ to 10^−9^ M) and BPs at different concentrations (10^−8^ to 10^−6^ M) for five days. The number of cells was measured using methylene blue staining. Briefly, after three PBS washes, the cells were fixed with 95% ethanol for 30 min and dried. The cells were then incubated in a methylene blue solution (1% in borate saline buffer) for 40 min and washed 3 times. To elute the stain, 0.1 M NaCl was added, and the absorbance at 620 nm was measured with an iMark Microplate Absorbance Reader (BioRad).

### BrdU incorporation assays

Two thousand cells were plated in 96-well plates and treated for 48 h with vehicle, E2 and BPs at different concentrations. After incubation for 1 h with BrdU, the cells were fixed in 90% ethanol/5% acetic acid and permeabilized in PBS/0.3% Triton. The cells were then incubated in 2 M NaCl at 37°C, followed by incubation in a 0.1 M Borate Buffer (pH 8.5). After a 30 min saturation step in PBS with 0.05% Tween20 and 2% Normal Donkey Serum, the cells were probed using the primary antibody anti-BrdU (Abcam, ab8152) at 37°C for 1 h. The secondary antibody used to target anti-BrdU was a Dylight 488 labeled anti-mouse IgG (Eurobio, 072-03-18-18) for 30 min at 37°C. Finally, a Hoechst staining was performed for 20 min at room temperature, and the results were analyzed using a Cellomics ArrayScan VTI HCS Reader (Thermo Fisher) in collaboration with the Impaccell technologic platform (Rennes 1 University, Rennes, France).

### Protein extraction/Western Blot

Whole cell extracts were prepared with 3× Laemmli buffer. A sonication step was performed before protein denaturation for 5 min at 95°C. The proteins were separated on SDS polyacrylamide gels and transferred onto polyvinylidene difluoride membranes (Millipore). The membranes were probed with specific antibodies, and the immunocomplexes were detected with a chemiluminescence system (Immun-Star, BioRad).

### Computer-simulated ligand binding (docking)

#### Protein input file preparation

A clean Estradiol receptor α input file (1ERE pdb, referred to in this paper as ER-E2) was generated using the protein preparation wizard from Maestro software (Maestro 8.5, academic campaign, Schrodinger website. Available: http://www.schrodinger.com, Accessed 2013 March 11). The protein was cleaned by removing water molecules, ligands and subunits b, c, d and f from the original ER-E2 pdb file. Next, the bond orders were assigned, and hydrogen atoms were added. The resulting receptor (ERα) was saved as a PDB file.

#### Ligand input file preparation

The ligand input structure was generated and 3D optimized with MarvinSketch Academic Package (MarvinSketch 5.10.0, 2012, ChemAxon website, http://www.chemaxon.com, Accessed 2013 March 11). The ligand structures were saved as a mol2 file.

#### GOLD docking protocol

For the study, the binding pocket of the receptor was defined from the crystallographic coordinates of the hydroxyl of estradiol. Dockings were performed using the GOLDScore fitness function under the 'Standard default settings' of GOLD software: a population size of 100, number of islands was 5, number of operations was 100,000, with a niche size of 2 and a selection pressure of 1.1.

### Statistical analysis

Statistical analysis was performed using Student's t-test. The values are provided as the mean ± standard error of the mean (SEM) and were considered statistically significant with p<0.05.

## Results

### Proliferative effects of benzophenone derivatives in ERα-positive MCF-7 breast cancer cells

**Figure 2:**
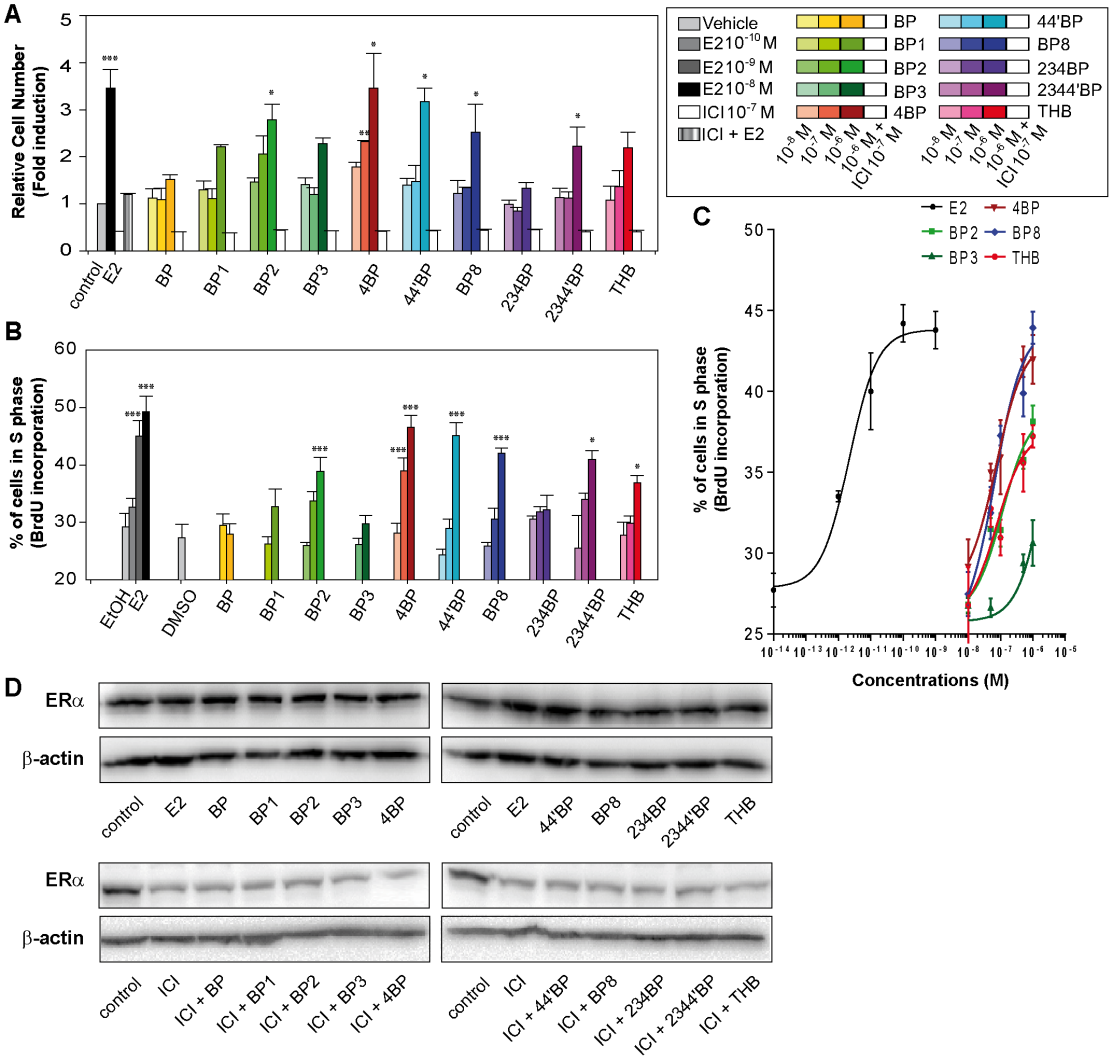
Proliferative effects of BPs in MCF-7 breast cancer cells. (**A**) After 48 h of steroid deprivation, MCF-7 cells were cultured in medium containing 2.5% dextran-treated charcoal stripped FBS and treated during 5 days with vehicle, 10^−8^ M estradiol (E2) or different concentrations of BPs (10^−8^, 10^−7^ and 10^−6^ M). In addition, cells were treated with 10^−7^ M of the anti-estrogen ICI_182,780_ (ICI) alone or in combination with 10^−8^ M E2 (hatched bar) or 10^−6^ M of each one of the BPs (open bars). Cell growth was evaluated using methylene blue assays and the results were expressed as fold induction between treated cells and vehicle-treated cells (considered as one-fold induction). (**B and C**) As in panel A, MCF-7 cells were cultured in medium containing 2.5% dextran-treated charcoal stripped FBS and treated with different concentrations of estradiol (10^−12^, 10^−11^, 10^−10^ M, illustrated by color gradations) or different concentrations of BPs (10^−8^, 10^−7^ and 10^−6^ M, illustrated by color gradations). The percentage of cells in S phase was evaluated 48 hours later using BrdU incorporation assays. Data are the mean values from triplicate experiments ± SEM (* P<0.05, **P<0.01, ***P<0.001). (**D**) Equal amounts of whole cell extracts from MCF-7 cells were loaded on denaturing gels. The ERα and β-actin protein levels were detected with specific antibodies as described in the Materials and Methods.

To characterize how BP derivatives impact the estrogenic response in breast cancer cell lines, we first screened the ability of BP derivatives to induce the proliferation of ERα-positive MCF-7 cells, which is controlled by estrogens. For this study, we chose ten BP derivatives that differ in the number and position of hydroxyl and/or methoxyl groups contained in their structures. Thus, we performed a 5-day proliferation assay, which allowed us to check the increase in cell number after treatment with different doses of BPs (10^−8^, 10^−7^ and 10^−6^ M; Fig. 2.A). As a control, we used 10^−8^ M E2, which induced a 3.5 fold increase in cell number after treatment. Of the 10 BPs tested, 6 were able to induce an augmentation in cell number. Although BP and 234BP had no significant impact on cell growth at the tested concentrations, BP2, 44′BP and 4BP at 10^−6^ M induced an increase in cell number comparable to the E2 effect (Fig. 2.A). Moreover, 4BP was able to induce a significant increase in cell number from 10^−7^ M. These proliferative effects of BPs are repressed when cells are cotreated with 10^−7^ M of the anti-estrogen ICI confirming that these effects are mediated by ERs, likely ERα as MCF-7 cells express mainly ERα. In addition, we performed a BrdU-incorporation assay to observe the BP-induced modulation of cell percentage in S phase (Figs. 2.B and C). The results from this approach were correlated with the BP-induced increase in cell number observed in the proliferation assays. Globally, the BPs can be divided into three groups with regard to their proliferative potentials: no or little activity (BP, BP1, BP3 and 234BP), medium activity (BP2, 2344′BP and THB), and high activity (4BP, 44′BP and BP8). As a control, we measured the level of ERα protein by Western Blot in untreated MCF-7 cells or cells treated with E2 (10^−8^ M) or ICI (10^−7^ M) and the different BPs (10^−6^ M) for 24 h. As shown in Fig. 2.D, the protein levels of ERα is drastically decreased after exposure to ICI but remained similar to the control after the exposure of MCF-7 cells to the different BPs. This indicates that proliferative effects observed upon BP treatments are not due to a consequence of changes in the level of intracellular ERα expression.

Altogether, these data are evidence of a proliferative effect of several BP derivatives in the MCF-7 ERα-positive breast cancer line, with the maximum mitogenic potential exhibited by 4BP (Table 2).

**Table 2:** Estrogenic activity of BPs on cell proliferation, endogenous E2-target gene stimulation and ER-mediated transcription at various ER-binding sites.

	Proliferation		Endogenous genes				Reporter genes				Interactions within ERα ligand binding site (H-Bond)
	Cell number	BrdU incorporation	CXCL12	Amphiregulin	pS2	PR	EREtk	Sp1	AP1	C3	
BP	–	–	–	–	–	–	–	–	–	–	Arg-394: 0; Glu-353: 0; Thr-347: 0
BP1	WA (≥10^−6^ M)	–	–	–	–	–	WA (≥10^−6^ M)	WA (≥10^−6^ M)	–	WA (≥10^−6^ M)	Arg-394: 1; Glu-353: 1; Thr-347: 0
BP2	Active (≥10^−6^ M)	WA (≥10^−6^ M)	–	WA (≥10^−6^ M)	WA (≥10^−6^ M)	–	Active (≥10^−7^ M)	Active (≥10^−7^ M)	–	WA (≥10^−7^ M)	Arg-394: 1; Glu-353: 1; Thr-347: 0
BP3	WA (≥10^−6^ M)	–	–	–	–	–	–	–	–	–	Arg-394: 0; Glu-353: 0; Thr-347: 0
4BP	Active (≥10^−7^ M)	Active(≥10^−7^ M)	Active (≥10^−7^ M)	Active (≥10^−6^ M)	Active (≥10^−6^ M)	Active (≥10^−7^ M)	Active (≥10^−7^ M)	Active (≥10^−6^ M)	–	WA (≥10^−6^ M)	Arg-394: 1; Glu-353: 1; Thr-347: 0
44′BP	WA (≥10^−6^ M)	WA (≥10^−6^ M)	–	WA (≥10^−6^ M)	Active (≥10^−6^ M)	Active (≥10^−6^ M)	WA (≥10^−6^ M)	Active (≥10^−6^ M)	–	WA (≥10^−6^ M)	Arg-394: 1; Glu-353: 1; Thr-347: 1
BP8	WA (≥10^−6^ M)	Active (≥10^−6^ M)	WA (≥10^−6^ M)	–	Active (≥10^−6^ M)	WA (≥10^−6^ M)	Active (≥10^−7^ M)	–	–	WA (≥10^−6^ M)	Arg-394: 0; Glu-353: 1; Thr-347: 0
234BP	–	–	–	WA (≥10^−6^ M)	–	–	WA (≥10^−6^ M)	–	–	–	Arg-394: 1; Glu-353: 2; Thr-347: 0
23′44′BP	WA (≥10^−6^ M)	WA (≥10^−6^ M)	–	WA (≥10^−6^ M)	–	–	WA (≥10^−6^ M)	–	–	WA (≥10^−6^ M)	Arg-394: 1; Glu-353: 2; Thr-347: 1
THB	WA (≥10^−6^ M)	WA (≥10^−6^ M)	–	WA (≥10^−6^ M)	Active (≥10^−6^ M)	–	Active (≥10^−7^ M)	Active (≥10^−7^ M)	–	WA (≥10^−7^ M)	Arg-394: 1; Glu-353: 1; Thr-347: 1

WA indicates weak activation, (-) indicates no effect.

### Differential activation of estrogen-target genes by benzophenone derivatives

**Figure 3:**
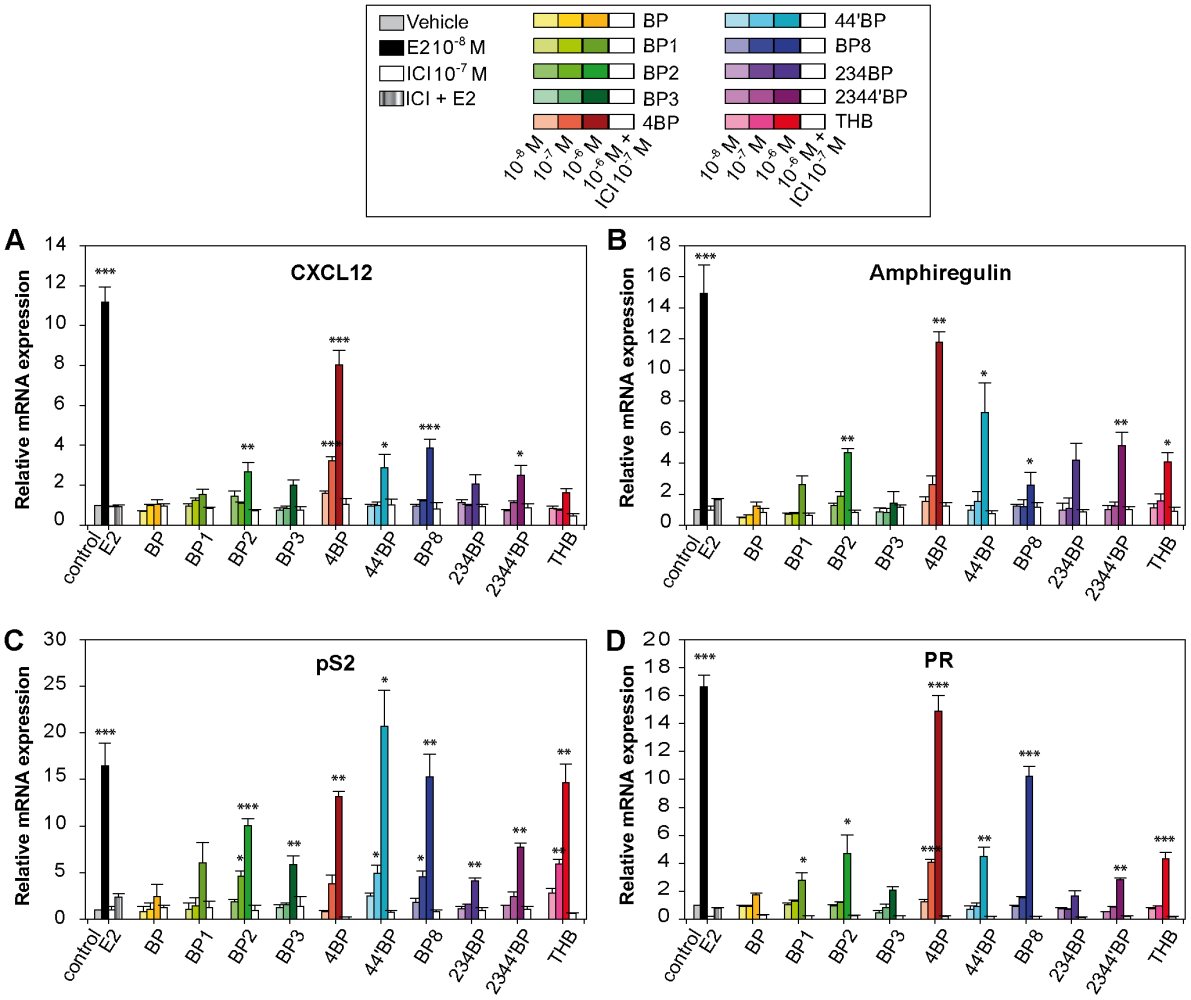
Evaluation of BP-induced expression of several endogenous estrogen-regulated genes. After hormone deprivation for 24 h, MCF-7 cells were grown in medium containing 2.5% dextran-treated charcoal stripped FBS and treated with 10^−8^ M E2 or different concentrations of BPs (10^−8^, 10^−7^ and 10^−6^ M, illustrated by color gradations) for 48 h. In addition, treatments to 10^−6^ M BPs were also performed in presence of 10^−7^ M of the anti-estrogen ICI_182,780_ (ICI) (open bars for BP + ICI treatments and hatched bar for E2 + ICI treatment). The expression levels of several E2-regulated genes, (**A**) CXCL12, (**B**) Amphiregulin, (**C**) pS2 and (**D**) Progesterone Receptor (PR), after the treatments were quantified using real-time PCR. Data are the mean values from triplicate experiments ± SEM (* P<0.05, **P<0.01, ***P<0.001).

As some BP derivatives are able to induce mammary cancer cell proliferation, we performed RT-PCR to assay the ability of BP derivatives to induce the expression of several estrogen-regulated genes, focusing on two genes that are tightly linked with estrogen-mediated proliferation, the CXCL12 and amphiregulin genes [20]–[22] (Figs. 3.A and B). Additionally, we also tested two classical estrogen-responsive genes, the pS2 (also known as trefoil factor 1, tff1) gene and Progesterone Receptor (PR) gene (Figs. 3.C and D). The results were obtained after 24 h of exposure of MCF-7 cells to increasing concentrations of each BP (10^−8^, 10^−7^ and 10^−6^ M). In addition, cells were also cotreated with 10^−6^ M BPs and 10^−7^ M ICI at the same time. All of the tested BPs failed to induce strong expression of CXCL12 gene, except 4BP. Nevertheless, BP2, 44′BP, BP8 and 2344′ significantly stimulated the expression of CXCL12, but the expression was ∼3 fold weaker amplitude than E2. The expression of the amphiregulin gene was significantly induced by six of the ten BPs, including THB that did not induce the CXCL12 gene. 4BP-induced expression of this gene was comparable with that of E2. Except for the parental BP, all BP derivatives resulted in at least a 4-fold induction of pS2 gene expression at a concentration of 1 µM. At this concentration, 4BP, 44′BP, BP8 and THB induced expression of pS2 in the same range as E2. For the PR gene, only 4BP and BP8 succeeded in inducing mRNA expression more than 10 fold relative to the control. In contrast, the treatment of the cells with BP1, BP2, 44′BP, 2344′BP and THB did not result in more than a 4–5 fold induction. For all the tested genes, the pure anti-estrogen ICI repressed the BP-mediated activation of E2-target genes, demonstrating that BP-induced transcription of these genes is mediated by ERs (Fig. 3).

To determine if a correlation exists between the transcriptional effect of BPs and their proliferative effect, we performed Pearson's tests. According to this test, a good correlation coefficient exists for both CXCL12 and amphiregulin gene inductions and cell proliferation (r_p_ = 0.8236 with a p-value = 0.00099 and r_p_ = 0.8845 with a p-value = 0.00013, respectively). In contrast, the induction of pS2 and PR genes exhibited poor correlation with BP-induced proliferation (r_p_ = 0.597 and 0.467 with p-values = 0.04 and 0.125, respectively).

Except for 4BP, which induced the expression of the four E2-target genes with a fold-induction comparable to that observed with estrogen, and the parental BP, which had no effect, the effects of the other BPs were more dependent on the tested genes. For instance, THB and 44′BP were found to be as potent as E2 in inducing pS2 expression, and they showed weak effects on the expression of the CXCL12, amphiregulin and PR genes. Although BP3 had weak estrogenic activity on the pS2 gene, it was inefficient in stimulating CXCL12, PR and amphiregulin gene expressions. These results suggest that the promoter context that controls the transcriptional activity of a specific gene plays an important role in the selective action of BPs.

**Figure 4:**
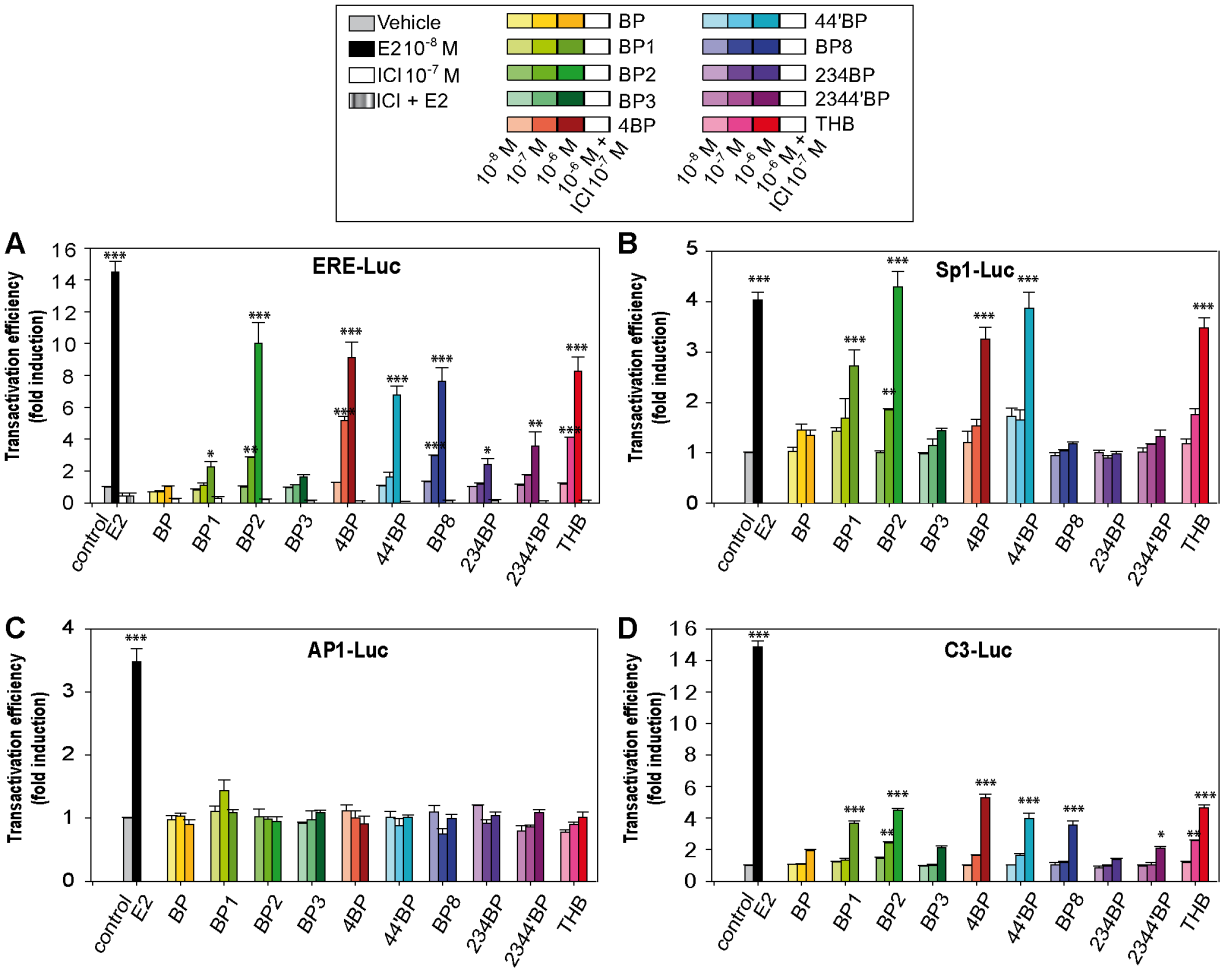
Estrogenic activity of BP derivatives using structurally different luciferase reporter assays in MCF-7 cells. Cells were transiently transfected with 100 ng of the reporter gene (ERE-Luc (**A**), Sp1-Luc (**B**), AP1-Luc (**C**) or C3-Luc (D) and 25 ng of the internal control CMV-βgal. Cells were treated for 36 h with vehicle, 10^−8^ M E2 or different concentrations of BPs (10^−8^, 10^−7^ and 10^−6^ M, illustrated by color gradations). In the panel A, transfected cells with the ERE-Luc were also treated with 10^−7^ M of the anti-estrogen ICI_182,780_ (ICI) alone or in combination with 10^−6^ M of each one of the BPs (open bars) or with 10^−8^ M E2 (hatched bar). Luciferase activities were normalized to β-galactosidase and expressed as the fold increase above vehicle alone. Each value represents the mean ± SD of at least three experiments (*P<0.05, **P<0.01, ***P<0.001, compared with control).

To further study this selectivity of BPs in transcriptional activation, we performed luciferase assays and examined the impact of BPs on the induction of several structurally different reporter genes (Fig. 4). ERα is able to enhance the transcription of estrogen-target genes by direct interaction with ERE or indirectly through complexes formed at Sp1- or AP1-binding sites [15]. Therefore, we used a reporter gene containing a consensus ERE (ERE-Luc), classically used to assay estrogenic potencies of EDCs, and reporter genes containing Sp1 or AP1 response elements (Sp1-Luc and AP1-Luc). Additionally, we tested the ability of BPs to induce the transcription of a complement 3-promoter containing reporter gene (C3-Luc), which is a reporter gene known to mainly exhibit sensitivity to AF1-dependent stimulation [19], [23]. After transfection, the cells were treated with E2 or increasing concentrations of BPs (10^−8^, 10^−7^ and 10^−6^ M) for 48 h, and then, the luciferase activities were determined. Except for BP and BP3, all the BPs were able to induce the activity of the ERE-Luc at a concentration of 1 µM, through an ER-dependent mechanism as these inductions were abolished by treatments with ICI. However, only BP2, 4BP, BP8 and THB exhibited significant activity at 0.1 µM (Fig. 4.A). Nevertheless, even if these four BP derivatives strongly induced the ERE- reporter gene, they did not reach the maximal transactivation efficiency as observed with 10^−8^ M E2. For the activation of Sp1-Luc, 1 µM of each of the following BP2, 4BP, 44′BP and THB were as potent as 10 nM E2. As observed with the ERE-Luc, BP, BP3, 234BP and 2344′BP were weakly active or not active on the Sp1-Luc reporter gene. In contrast, BP8, which was among the most estrogenic compounds with regard to the ERE-Luc reporter gene, was inefficient in the activation of Sp1-Luc (Fig. 4.B). On the other hand, BP1 and 44′BP were more effective on Sp1-Luc than on ERE-Luc. Surprisingly, none of BP derivatives were found to exhibit estrogenic activities on the AP1-Luc reporter gene (Fig. 4.C). Finally, the transcriptional response of the C3-Luc reporter gene resulted in a profile comparable with the ERE-Luc, but with lower fold inductions than those obtained with E2 treatment (Fig. 4.D). This result suggests that the estrogenic effects of BPs are mainly due to the activation of the AF-2 transactivation function of ERα.

Collectively, these data confirm the estrogenicity of BPs at the transcriptional level and indicate that BPs possess a selectivity of action depending on the promoter contexts and on estrogen-target genes despite their similar structures (Table 2). These observations suggest that the structure of these compounds, especially the hydroxyl groups, is central in defining the estrogenic targets at the transcriptional level and the amplitude of the estrogenic response.

### Docking analysis of BP derivatives in the binding site of ERα

**Figure 5:**
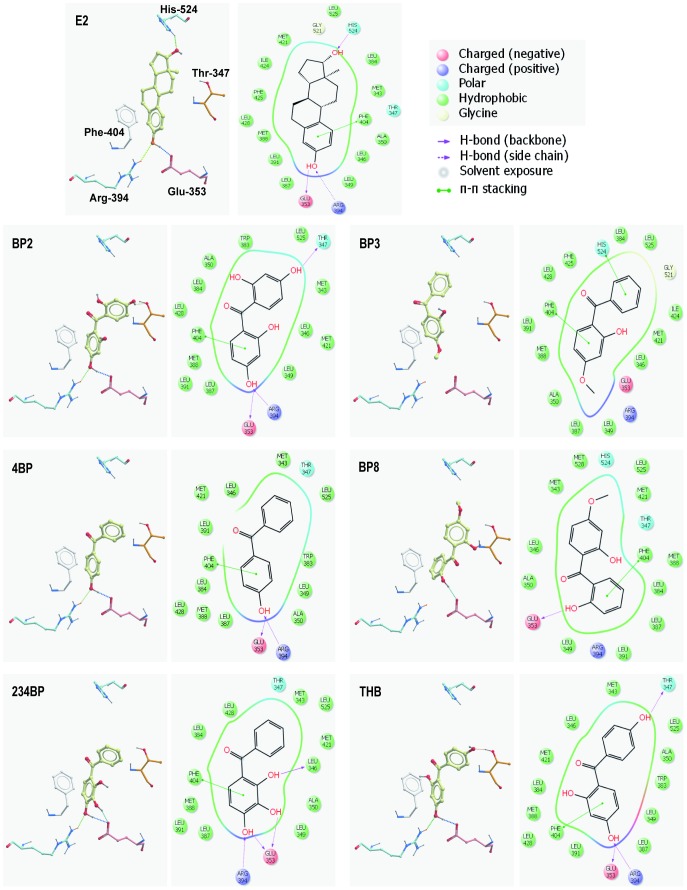
Docking views of BPs in the ERα ligand binding pocket. 3D and 2D docking views of E2, BP2, BP3, 4BP, BP8 and 234BP are shown. For the 3D views, only the H-bonds are shown, whereas the 2D views show H-bonds and π-π interactions. Phe: Phenylalanine, Met: Methionine, Arg: Arginine, Leu: Leucine, His: Histidine, Thr: Threonine, Glu: Glutamic acid, Ile: Isoleucine, Ala: Alanine, Gly: Glycine.

To better understand the selective estrogenic activities of BPs, we investigated how BPs interact in the ligand binding pocket of ERα, as the addition of hydroxyl groups is known to facilitate hydrogen bonding. Indeed, improper interactions of these compounds could be able to favor a different conformation of ERα from the E2-induced conformation, resulting in slight differences in the biologic effects. The dockings of six representative BP derivatives in the binding site of ERα, as well as the docking of E2, are shown in Fig. 5.

**Figure 6:**
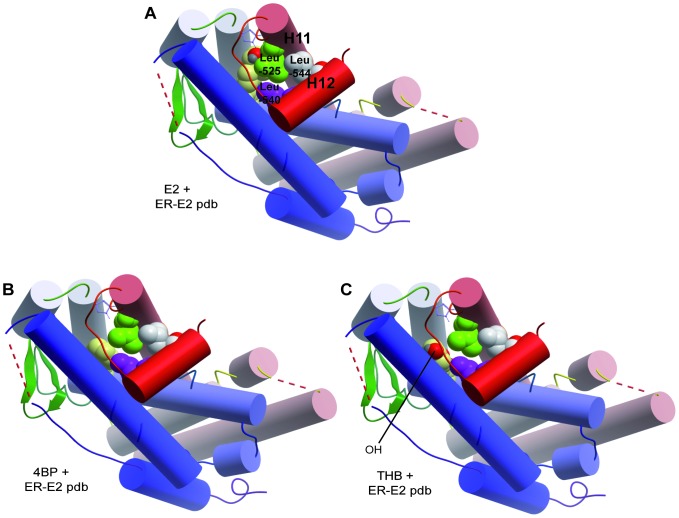
Cylinder representations of the interaction of E2, 4BP and THB in the E2-liganded-ERα crystal structure. The Leu-525 (in green) of helix 11, and Leu-540 and Leu-544 (in purple) of helix 12 are shown. In the correct ER 3D structure, these leucines interact with E2 through hydrophobic bonding, stabilizing the structure of helix 12 in an activated form. With 4BP, these interactions are still possible, whereas with THB, the hydroxyl group (in red) can interfere and destabilize the structure. Arg: Arginine, Leu: Leucine, His: Histidine, Thr: Threonine, Glu: Glutamic acid.

In the ER crystal structure, E2 establishes Hydrogen-bonding (H-bond) interactions between its A-ring and the Glu-353 and Arg-394 from ERα. An additional H-bond between the D-ring and His-524 stabilizes the structure. With most of the BPs, only the H-bonds with Glu-353 and Arg-394 are maintained and the H-bond with His-524 is lost. If the second aromatic ring of the BPs is hydroxylated in the correct position, an alternative H-bond with Thr-347 (within helix 3) can form, as observed with BP3 and THB. Moreover, van der Waals interactions between the hydrophobic residues in the ligand-binding pocket of ERα (Fig. 5, top 2D structure) and the phenyl rings of E2 creates a favorable surface (helix 12) in the ligand-binding pocket of ER that allows it to interact with transcriptional coactivators. Hence, in a proper agonist-conformation of the ER 3D structure, the Leu-540 (within helix 12) and Leu-525 (helix 11) interact by hydrophobic bonding, stabilizing the structure of helix 12 in an activated form (Fig. 6.A). Interestingly, our analysis indicates that BP derivatives without a hydroxyl in the second aromatic ring, such as 4BP, could increase the hydrophobic bonding and favor the proper positioning of helix 12 (Fig. 6.B). Inversely, a BP derivative with a polar hydroxyl in the second aromatic ring, such as THB, could decrease the hydrophobic bonding and cause improper positioning of helix 12 due to steric hindrance (Fig. 6.C).

## Discussion

BP and its hydroxylated derivatives are widely used in industry as UV filters for sunscreens, inks and plastic packaging. In this study, we analyzed the estrogenic potencies of ten UV filters from the BP family on several biological responses induced by estrogens, including proliferation, transcription and binding to the estrogen receptor. This study was conducted *in vitro* in a mammary cancer cell context because very few studies have demonstrated the estrogenic impact of BPs on breast cancer with regard to proliferation. Furthermore, the comparison of the estrogenic activities of BPs with very close structures that only differ in the position and number of hydroxyl or methoxyl groups highlights the importance of the ligand structure for the specificity of the estrogenic response.

In light of our results, some BPs were able to exhibit estrogenic activities in MCF-7 cells, including proliferation, at concentrations of the order of micromolar and lower (Table 2). To our knowledge, this is the first study reporting the proliferative effects of these 10 BP derivatives, as well as their estrogenic effect on structurally different E2-target genes. Of the 10 BPs, 6 were able to significantly stimulate the proliferation of MCF-7 breast cancer cells. Listed in order of decreasing potency, they are 4BP>44′BP>BP8>2344′BP>BP2>THB. In contrast, BP, BP1, BP3 and 234BP exhibited very low or no induction of proliferation. In agreement with several other reports [24], [25], we found that BP3, one of the most studied and common BPs, cannot induce proliferation or E2-dependent transcriptional activity. However, studies of BP3 metabolism in rats have revealed that BP3 can be converted into at least three metabolites that exhibit estrogenic potencies, BP1, BP8 and THB [24], [26]. Our results indicate that BP-induced CXCL12 and amphiregulin gene expression correlates well with the BP proliferative effects, indicating that analysis of these genes could be an alternative and early method for the assessment of the proliferative impact of estrogenic compounds. This is also in good agreement with our previous reports [20], [31]. In contrast, we have found very poor correlation between the proliferation and expression of the commonly used E2-target genes pS2 and PR, which are not directly involved in the processes of proliferation. Thus, ER-mediated regulation of CXCL12 and amphiregulin could involved molecular mechanisms distinct from pS2 and PR, explaining a better correlation of CXCL12 and amphiregulin expression with cell proliferation.

In addition, our study highlights the necessity to perform several tests to clearly characterize the estrogenicity of compounds. Numerous tests have been developed to assay the estrogenic potencies of environmental contaminants based on whole organisms [27], [28], cell systems [29]–[31] or biochemical approaches [32]. However, one type of test is likely not sufficient to define a xenoestrogen, as various ligands can exhibit species- and cell type-specific activities and result in differential recruitment of the ER and cofactors [33]. Thus, estrogenic abilities based on one parameter may not reflect a physiological response, as exemplified by the 2344′BP- and THB-induction profile of the pS2 gene or the ERE-Luc reporter gene that do not result in a corresponding proliferation profile.

Our examination of the effect of BP derivatives on ER-mediated transcription at different ER-binding sites showed that a great diversity exists in the ligand responsiveness on E2-target genes and depends on the ER-binding site properties. Although the reporter gene containing an ERE sequence was the most responsive gene to BP derivatives, none of these chemicals were able to activate the AP1 promoter. Moreover, regarding the C3 promoter, the BP derivatives showed weak estrogenic activity and none acted as a full agonist, suggesting that only the AF2 transactivation function of the receptor is activated. Collectively, these data suggest that different types of ER-binding sites within endogenous gene promoters may be responsible for the differential activation of E2-target genes by BPs. Accordingly, because the ER-binding sites are decisive parameters in the transactivation potency of ER bound BP derivatives, some of these chemicals may be used as a selective ligand to differentially activate E2-target gene populations. However, further investigation is necessary to describe the molecular mechanisms and the molecular pathways involved in these phenomena in more detail.

Confirming observations from another team [25], we found that the core BP molecule has no estrogenic activity, and addition of a hydroxyl group on one of the phenyl rings is essential for a maximum estrogenic activity, as exemplified by 4-hydroxy-benzophenone (4BP), which exhibits estrogenic potency and differs from BP by one hydroxyl group (Table 2). Indeed, docking experiments highlighted the necessity of a hydroxyl group to permit the proper interaction of the BPs in the ERα ligand-binding pocket (Fig. 5). Additional hydroxyl or methoxyl groups could alter the way molecules interact in the ligand-binding pocket, altering subsequent biological responses. A hydroxyl group on carbon 4 of the phenyl A-ring is ideal because it allows BPs to interact with Arg-394 and Glu-353, enhancing stability between the BPs and ERα. However, our docking analysis suggests that some BPs, such as BP8, which has a hydroxyl group on carbon 2, can only interact with Glu-353 *via* its OH group, which may create a weaker interaction, explaining the variability observed in its estrogenic capability. On the other hand, BP3 possesses a hydroxyl group on carbon 2 of the phenyl ring that also carries a methoxyl group on carbon 4, and BP3 exhibits very little estrogenic activity as compared to other BPs tested in our study. These results are also consistent with our docking experiments that failed to find a stable orientation and position of BP3 in the ligand binding pocket of ERα. This could be due to steric hindrance induced by the methoxyl group of BP3 or the intramolecular bonding between the hydroxyl group on carbon 2 and the carbonyl group. In addition, OH groups on the B-ring could also establish H-bonds with Thr-347, inducing changes in the 3D structure of the ERα and contributing to the differential action of the BP derivatives. These interactions have been recently described for two major environmental pollutants, bisphenol A [34] and polychlorinated biphenyl (PCB) [35].

The interaction of E2 with ERα induces an agonist conformational change in the ligand-binding pocket of the receptor, which was reported to be critical for accurate positioning of helix 12 and, consequently, for the transactivation function of AF2. Based on the structural studies, the phenyl ring of E2 interacts with Leu-540 (helix 12) and Leu-525 (helix 11) through hydrophobic bonding. Moreover, the presence of E2 in the ligand-binding pocket forces Leu-544 (helix 12) and Leu-525 to interact through van der Waals interactions, forming a stable and suitable conformation. Thus, OH groups in the phenyl B-ring of BPs may create a repulsion force which constrains helix 12 in an unfavorable position. Interestingly, our modeling suggests that 4BP could favor hydrophobic interactions. In contrast, the hydroxyl group of the phenyl B-ring of THB could disrupt either the hydrophobic bonding with Leu-540 and Leu-525 or the van der Waals interactions between Leu-525 and Leu-544. For instance, the selective ER modulator (SERM) Lasofoxifene was reported to induce a different rotamer of Leu-525 due to the close contact between Leu-525 and an oxygen atom in the compound, abolishing van der Waals interactions between Leu-525 and Leu-544 [36]. Therefore, based on differences in the residues of the ERα ligand-binding site, BPs could lead to slightly different ligand-dependent conformational changes of the activated receptor, which could alter the receptor's ability in cofactor recruitments, transcriptional regulation and cell response.

Only a few BP UV filters have been investigated in the environment to date. However, several BPs have been found at different concentrations in several matrixes, notably in aquatic environments including lakes, wastewater or swimming pool water, as well as in sediment, sewage sludge and soil (Table 1). In addition, BPs have also been detected in numerous biological samples, such as fish fat, urine or milk [2]. Hayashi and coworkers have demonstrated that BP can be converted into 4BP after exposure to sunlight [37]. This finding is consistent with the potential danger associated with using a BP-containing sunscreen that can produce highly estrogenic compounds that are in direct contact with the skin. BPs are highly lipophilic and are assumed to bioaccumulate in humans and wildlife [38]. Moreover, during the summer, a large portion of skin (up to 75%) is treated daily or twice a day with sunscreens [39], and these UV filters are absorbed by the skin and enter the human body [40], [41]. Considering our results, direct application of BP-based sunscreen on the breast and the subsequent skin absorption could favor the proliferation of ERα-positive epithelial cells, increasing the probability of developing a breast cancer or stimulating the growth and progression of a pre-existing tumor. A recent study has revealed a potential association between the urinary concentration of some BP derivatives and another estrogen-dependent disease, endometriosis [42]. Nevertheless, more studies should be conducted to shed light on the possible correlation between BP exposure and breast cancer.
